# The Autism–Psychosis Continuum Conundrum: Exploring the Role of the Endocannabinoid System

**DOI:** 10.3390/ijerph19095616

**Published:** 2022-05-05

**Authors:** Marco Colizzi, Riccardo Bortoletto, Rosalia Costa, Sagnik Bhattacharyya, Matteo Balestrieri

**Affiliations:** 1 Unit of Psychiatry, Department of Medicine (DAME), University of Udine, 33100 Udine, Italy; matteo.balestrieri@uniud.it; 2 Department of Psychosis Studies, Institute of Psychiatry, Psychology and Neuroscience, King’s College London, London SE5 8AF, UK; sagnik.2.bhattacharyya@kcl.ac.uk; 3 Child and Adolescent Neuropsychiatry Unit, Maternal-Child Integrated Care Department, Integrated University Hospital of Verona, 37126 Verona, Italy; riccardo.bortoletto@studenti.univr.it; 4 Community Mental Health Team, Friuli Centrale University Health Service (ASUFC), 33057 Palmanova, Italy; rosalia.costa@asufc.sanita.fvg.it

**Keywords:** neurodevelopment, mental health, delta-9-tetrahydrocannabinol, cannabidiol, cannabis

## Abstract

Evidence indicates shared physiopathological mechanisms between autism and psychosis. In this regard, the endocannabinoid system has been suggested to modulate neural circuits during the early stage of neurodevelopment, with implications for both autism and psychosis. Nevertheless, such potential common markers of disease have been investigated in both autism and psychosis spectrum disorders, without considering the conundrum of differentiating the two groups of conditions in terms of diagnosis and treatment. Here, we systematically review all human and animal studies examining the endocannabinoid system and its biobehavioral correlates in the association between autism and psychosis. Studies indicate overlapping biobehavioral aberrancies between autism and schizophrenia, subject to correction by modulation of the endocannabinoid system. In addition, common cannabinoid-based pharmacological strategies have been identified, exerting epigenetic effects across genes controlling neural mechanisms shared between autism and schizophrenia. Interestingly, a developmental and transgenerational trajectory between autism and schizophrenia is supported by evidence that exogenous alteration of the endocannabinoid system promotes progression to inheritable psychosis phenotypes in the context of biobehavioral autism vulnerability. However, evidence for a diametral association between autism and psychosis is scant. Several clinical implications follow from evidence of a developmental continuum between autism and psychosis as a function of the endocannabinoid system dysregulation.

## 1. Introduction

In the absence of clear neurobiological markers of disease, the current identification of psychiatric disorders relies on a phenotypical classification system which struggles to consider the large disorder comorbidity and the significant symptom heterogeneity [1]. While acknowledging the usefulness of grouping ostensibly recurrent symptoms within a unique condition, it is not surprising that such boundaries between psychopathological phenomena have been questioned for over a century [2]. This is particularly relevant to conditions with onset in the neurodevelopmental period in which the last five decades have seen several twists and turns in bringing order into potentially arbitrary distinct clinical phenotypes that may reflect a single disease process [3].

Two groups of conditions scientifically put to the stand are autism and schizophrenia, with the former progressively emancipating from the latter since its first introduction as a schizophrenia-related impairment [4]. In fact, a diagnostic incompatibility with schizophrenia was proposed when autism debuted in the Diagnostic and Statistical Manual of Mental Disorders in 1980 [5]. However, such separation has been experiencing the setbacks of recent neurobiological advances, making the differential physiopathological mechanisms leading to the two disorders progressively more nuanced [6]. Biochemical, structural, genetic, physiological, and pathophysiological studies have proven shared abnormalities of interconnected systems of genes and molecular pathways regulating brain specialization during the embryonic period [7]. In addition, evidence converges on a correlation between the nature and severity of the embryonic alteration on one hand and the earliness and severity of the behavioral pathology on the other, in line with the so-called neurodevelopmental gradient hypothesis [8]. Therefore, various clinical presentations may be possible; this is further complexified by continuing brain maturation and bidirectional environmental conditioning over the lifespan, especially until neurodevelopment is complete [9]. To acknowledge the neurodevelopmental continuum model [10] stemming from the evidence that childhood-onset (e.g., autism) and adolescence- and early adulthood-onset psychiatric disorders (e.g., schizophrenia) fall on a physiopathological continuum [11], recent diagnostic revisions have included the newly termed autistic and schizophrenia spectrum disorders, reflecting the higher validity and reliability of a more dimensional approach [12]. In the search for neurobiological commonalities between the two groups of conditions, particular attention has been given to aberrancies in biological systems involved in synaptic development, function, and plasticity, which are frequently identified in patients diagnosed with either schizophrenia or autism [13,14,15]. In this regard, the endocannabinoid (eCB) system has been found to play a crucial role in regulating synaptic plasticity and the functioning of multiple neural networks, not only in the mature nervous system [16,17,18] but also in the immature nervous system while developing, with long-lasting consequences [19,20,21,22,23]. Recent reappraisals of the literature suggest potential implications of the eCB system in the pathogenesis of several neuropsychiatric disorders including but not limited to schizophrenia and autism spectrum disorder [24].

### Objectives

Due to its role in modulating both the structure and function of neural circuits during the early stage of neurodevelopment, the eCB system could represent a promising neurobiological mechanism common to autism and psychosis. Studies in this area may help to improve our understanding of neurodevelopment as well as support reasonable medical use of eCB-related drugs in the longer term from a preventative perspective. This systematic review aims to bring together and discuss all available data generated by clinical and preclinical studies, investigating the role of the eCB system in neurodevelopment, with particular attention to the diagnostic conundrum of differentiating autism and schizophrenia spectrum disorders. All interventional and observational studies will be reviewed, employing both retrospective and prospective methodological approaches with any eCB-related biobehavioral markers.

## 2. Experimental Procedures

### 2.1. Inclusion and Exclusion Criteria

In order to summarize previous evidence on the subject, inclusion criteria for studies were as outlined below: (1) human or animal studies; (2) studies assessing autism and psychosis neurobiological features as a function of the endocannabinoid system modulation; (3) studies assessing autism and psychosis socio-behavioral traits as a function of the endocannabinoid system modulation; and (4) studies assessing autism and psychosis genetic background as a function of the endocannabinoid system modulation. Exclusion criteria were as follows: (1) studies evaluating both autism and psychosis independently from the endocannabinoid system; (2) studies investigating autism but not psychosis through the endocannabinoid system; and (3) studies investigating psychosis but not autism through the endocannabinoid system.

### 2.2. Search Strategy and Data Extraction

A literature search was conducted using electronic databases (Pubmed, Web of Science and Scopus) for any published original study written in English, using a combination of search terms describing and/or concerning the endocannabinoid system (‘cannab*’, ‘delta-9-tetrahydrocannabinol’, ‘Δ-9-tetrahydrocannabinol’, ‘marij*’, ‘marih*’, ‘sativa’, ‘indica’), autism spectrum disorder (autis*, asperger), and psychosis (‘psycho*’, ‘schizophreni*’) on 3 April 2022. The broad meaning of terms used was intended to make the study search as inclusive as possible. Reference lists of eligible studies were screened to identify additional eligible research. Publication data screening and extraction were performed following a two-step selection process (conventional double-screening) conducted by two reviewers independently of each other (R.B. and M.C.).

### 2.3. Risk of Bias

Considered the methodological heterogeneity of the studies (Table 1a,b) included in this review, a reasonably inclusive and flexible approach was deemed as appropriate to perform risk of bias and study quality assessments. For this purpose, an adapted set of criteria suggested by the Agency for Healthcare Research and Quality (AHRQ) guidance was used for interventional and observational studies in humans. Additionally, factors possibly accounting for similarities and differences between animal studies were assessed, extracting information about study characteristics, including animal model (mouse or rat), developmental stage (postnatal, adult), gender, endocannabinoid system involvement (i.e., interventional administration, assessment in tissues, gene expression), autism involvement (i.e., idiopathic, genetically or inflammatory-induced models, genetic liability), and psychosis involvement (i.e., behavioral features, genetically or inflammatory-induced models, genetic liability) (Table 2a,b). 

## 3. Results

### 3.1. Study Selection

**Figure 1:**
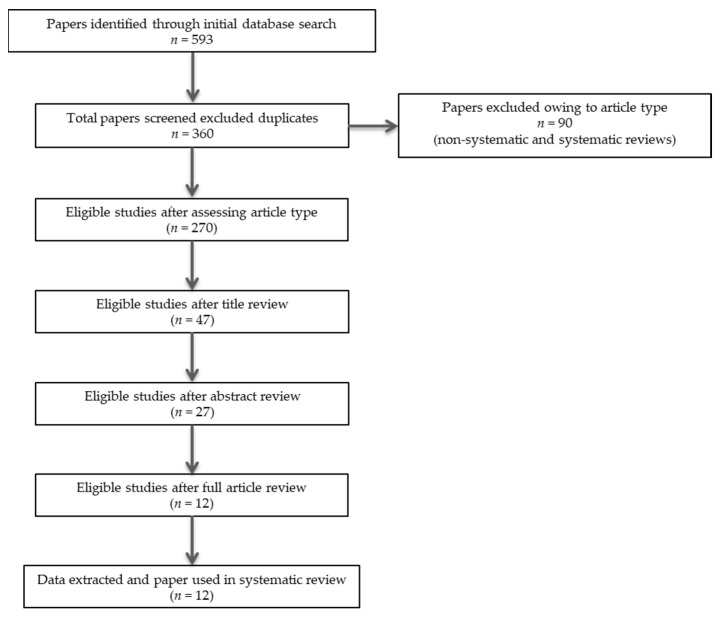
PRISMA flowchart of search strategy for systematic review.

In summary, 360 records were retrieved. After excluding articles owing to article type (systematic and non-systematic reviews), by using a three-step screening approach, titles, abstracts, or full texts of all records were screened against the inclusion and exclusion criteria (Figure 1). A final list of twelve studies (six human, four animal, and two using both humans and animals) was used for systematic analysis in this review (Table 1). In total, the eligible studies provided different types of evidence for a role of the endocannabinoid (eCB) system in the continuum between autism and schizophrenia spectrum disorders (Table 1). These included: (i) evidence for a diametral relationship between autism and psychosis-related phenotypes as a function of the eCB system (two studies); (ii) evidence for an overlapping relationship between autism and psychosis-related phenotypes as a function of the eCB system (six studies); and (iii) evidence for a developmental trajectory between autism and psychosis-related phenotypes as a function of the eCB system (seven studies). Additional data on methodological quality of studies are reported in Table 2. A brief synthesis of the main results is presented below and summarized in Table 1.

### 3.2. Evidence for a Diametral Relationship between Autism and Psychosis-Related Phenotypes as a Function of the eCB System Modulation

The current review identified only very limited evidence supporting a diametral relationship between autism and schizophrenia spectrum disorders based on diametrically opposite responses to the eCB system manipulation. It included a preclinical [25] and a clinical study [32]. The animal study explored the effect of disrupting the eCB system in the BTBR T+tf/J mice, a preclinical model exhibiting autism-like behavioral phenotypes [25]. Interestingly, delta-9-tetrahydrocannabinol (THC) reduced the aberrant enhanced basal locomotor activity observed in BTBR, while enhancing locomotor activity in the C57BL/6J and S129 mice which were the control background mice for the BTBR animals. In addition, the reduced immobility time and increased immobility count observed in BTBR animals was not modulated by THC, which instead dose-dependently modulated such behavior in control animals. Finally, the cerebellar gene expression of the mouse cannabinoid receptor type 2 transcript CB2A was found to be upregulated in BTBR mice.

The human study investigated the effect of cannabidiol (CBD)-rich medical cannabis in 60 children with severe autism spectrum disorders (ASD) [32]. Despite being generally therapeutic, treatment with CBD-rich medical cannabis resulted in four children (8%) requiring a medication adjustment (i.e., more psychopharmacological treatments or higher dose) to support clinical stability. In addition, almost 50% of the patients were rated to have insufficient response and switched to strains with a relatively high THC concentration (CBD:THC ratio from 20:1 to 6:1) to ameliorate symptom severity.

### 3.3. Evidence for an Overlapping Relationship between Autism and Psychosis-Related Phenotypes as a Function of the eCB System Modulation

Half of the evidence gathered in this review supports neurobiological mechanisms common to autism and schizophrenia spectrum disorders, with reference to the eCB system. The first of these studies explored the β-neurexin knockout (KO) phenotype as a potential preclinical model carrying alterations that have being implicated in both autism and schizophrenia [26]. The study found that β-neurexin KO results in an increased basal eCB signaling, which in turn decreases Ca^2+^-influx and glutamate release at excitatory synapses and blocks long-term plasticity. Such mechanism was reported to be behaviorally important, as knockout of β-neurexins in CA1-region neurons resulted in impaired contextual fear. Long-term plasticity appeared to be restored by cannabinoid receptor type 1 (CB1) inhibition (i.e., blocking presynaptic eCB signaling) or 2-arachidonoylglycerol (2-AG) synthesis inhibition (i.e., hampering postsynaptic 2-AG release). Using a similar approach, another study investigated impaired social behavior as a phenotype associated with both autism and schizophrenia [27]. It used a preclinical model of early life lipopolysaccharide (LPS)-mediated inflammation (postnatal day 14) to induce adolescent aberrant sociability and related neurobiological alterations (postnatal day 40). In adolescence, animal models presented with reduced CB1 binding site density and increased fatty acid amide hydrolase (FAAH, the enzyme which metabolizes the eCB anandamide, AEA) activity as well as relatively increased AEA concentrations. Oral FAAH inhibition, which selectively increases AEA, restored social behavior, with FAAH inhibition trough infusion in the basolateral amygdala being sufficient to normalize behavior in females.

A different line of research suggested a role of CBD in both autism and schizophrenia by studying the epigenomic activity of protracted CBD exposure [30]. The study found that CBD induces methylation changes in adult mice hippocampus in 3323 differentially methylated loci (DMLs), with a small skew toward global hypomethylation. Gene ontology enrichment analysis revealed genes involved in cell adhesion and migration, dendritic spine development, and excitatory postsynaptic potential. Further, to evaluate DML enrichment in the context of disease, genes containing DMLs were compared to the Human Mouse Disease Connection (HMDC) database, revealing an overrepresentation of DMLs in gene sets associated with autism spectrum disorder and schizophrenia, ranking first and third, respectively. Interestingly, the human study supporting potentially diametral responses to CBD-rich medical cannabis in ASD children also reported behavioral outbreaks that were much improved or very much improved in 61% of patients [32]. Preparations with high CBD:THC ratio (20:1) were well tolerated, with no significant adverse effects. In addition, 33% of patients received less medications or lower dosage and 24% of patients stopped taking medications because of substantial improvement.

A different type of study conducted gene-based tests of association to identify genetic risk variants of cannabis use [31]. Four protein-coding genes and one intergenic region were significantly associated with lifetime cannabis use, suggesting it to be a highly polygenic trait: (i) neural cell adhesion molecule 1 (NCAM1, on 11q23), which is part of the NCAM1–TTC12–ANKK1–DRD2 (NTAD) gene cluster, with relevance for neurogenesis and dopaminergic neurotransmission; (ii) cell adhesion molecule 2 (CADM2, on 3p12), belonging to the immunoglobulin (Ig) super-family and previously associated with autism spectrum disorders; (iii) short coiled-coil protein (SCOC, on 4q31), possibly involved in important biological functions, such as the regulation of gene expression through the regulation of transcription factor binding; (iv) SCOC antisense RNA1 (SCOC-AS1, on 4q31); and (v) potassium channel, subfamily T, member 2 (KCNT2, on 1q31). Complimentary evidence of common genetic background to autism, schizophrenia, and cannabinoid-mediated behavior was recently provided by a study of induced dopaminergic neurons (iDANs) [36]. Along with bipolar disorder, specifically expressed genes (SEGs) in iDANs were reported to be enriched for cannabis use disorder (CUD), ASD, and schizophrenia. CUD, bipolar disorder, and schizophrenia risk loci were also enriched in induced GABAergic neurons and glutamatergic neurons.

### 3.4. Evidence for a Developmental Trajectory between Autism and Psychosis-Related Phenotypes as a Function of the eCB System Modulation

Most studies included in this review provided evidence for a developmental trajectory between autism and psychosis-related phenotypes, thus supporting the possibility of comorbidity. While supporting both diametral and overlapping relationships between autism and schizophrenia spectrum disorders, the already mentioned study of CBD-rich medical cannabis use in severe ASD also found that strains with a relatively high THC concentration (CBD:THC ratio, 6:1) are associated with a serious psychotic episode requiring treatment with an antipsychotic [32]. Due to the limited sample power and participants using various cannabis strains from different growers and a broad range of CBD and THC doses, no further enquiries regarding the psychosis-inducing effects of cannabis in ASD were possible. However, more recent anecdotal evidence indicates that cannabis use may result in manic and/or psychosis symptoms in ASD, which may persist even following cannabis discontinuation and antipsychotic initiation [35].

In another line of research, relative to vehicle treatment, acute THC exposure was reported to significantly alter 497 genes in human-induced pluripotent stem cells (hiPSCs), with chronic THC exposure perturbing up to 810 genes. Altered transcripts following THC exposure were found in a substantial number of genes linked to autism (80 genes) and intellectual disability (167 genes), with fewer overlapping with schizophrenia [33]. Interestingly, THC-treated neurons displayed significant synaptic, mitochondrial, and glutamate signaling alterations that resembled those observed in schizophrenia hiPSC- derived neurons. Thus, autism-related genes seemed to be prominently involved in THC signaling, with schizophrenia risk resulting from THC-mediated activity-dependent pathway disruption. In a further study, the developmental trajectory between autism and schizophrenia was independently supported by genetic evidence that autism-related risk genes increase the liability for lifetime cannabis use, with implications for dysfunctional dopamine signaling control [31]. Additional evidence of a genetic link between autism and schizophrenia through the eCB system was offered by a genetic-association study of psychotic experiences among a population-based cohort [34]. By conducting analyses of polygenic risk scores (PRS), psychotic experiences were found to be associated with genetic liability for several major psychiatric disorders, including ASD and schizophrenia. In addition, individuals reporting psychotic experiences had an increased burden of Copy Number Variations (CNVs) associated with schizophrenia and neurodevelopmental disorders more widely. Finally, loci identified through Genome-Wide Association Studies (GWAS) included a locus in Ankyrin-3 (ANK3), which has been implied in ASD, and a locus in cannabinoid receptor type 2 gene (CNR2), which has been particularly associated with distressing psychotic experiences.

The strongest evidence for a developmental association between autism and schizophrenia was provided by recent studies exploring the neurodevelopmental consequences of exposure to cannabis and THC in both humans and animals [28,29]. The Discs-Large Associated Protein 2 (DLGAP2), which is involved in synapse organization and neuronal signaling and has been strongly implicated in autism, was found to be hypomethylated in cannabis users’ sperm as compared with non-users. In addition, the higher the methylation in the human brain, the lower was the mRNA expression. Importantly, animal data indicated an intergenerational inheritance of altered DNA methylation pattern in DLGAP, with hypomethylation in the sperm of THC-exposed rats compared to controls as well as hypomethylation in the same DLGAP region in the nucleus accumbens of the offspring [28]. Further, THC exposure induced an altered methylation pattern in seven neurodevelopmentally active genes in rat sperm (hypermethylation: Leucine Rich Repeat Transmembrane Neuronal 4 (LRRTM4); hypomethylation: Discs Large MAGUK Scaffold Protein 4, (Dlg4), SH3 and Multiple Ankyrin Repeat Domains 1 (Shank1), Glutamate Ionotropic Receptor Delta Type Subunit 1 (Grid1), Neurexin 1 (Nrxn1), Neurexin 3 (Nrxn3), and Synaptotagmin 3 (Syt3)), affecting several Biological Process Gene Ontology (GO) terms involved in neuronal development. Human data confirmed functional interactions between these seven genes, with relevance for Biological Process GO terms such as social behavior, vocalization behavior, and learning. Interestingly, many autism candidate genes presented with bivalent chromatin markings, indicating that they are inherently vulnerable to disruption of DNA methylation and potentially altered expression because of environmental exposure [29].

## 4. Discussion

This is the first systematic review of all studies investigating the role of the endocannabinoid (eCB) system in the connections between autism and schizophrenia spectrum disorders in humans and animals. Previous reviews of both preclinical and clinical evidence have mainly reappraised the eCB signaling in the two conditions separately, indicating that alterations of the eCB system at multiple levels are critical to both autism [37] and schizophrenia [38]. In addition, accumulating evidence has been suggesting a role of the eCB system modulation as an innovative therapeutic strategy against either autism [39,40] or schizophrenia [41,42] spectrum disorders. 

Overall, this review demonstrated shared biobehavioral aberrancies between autism and schizophrenia, subject to correction by modulation of the eCB system [26,27], and provided evidence of common cannabinoid-based pharmacological strategies for the treatment of both conditions [32], thus suggesting a potential dimensional approach to their management [43]. Intriguingly, cannabinoid-based treatment effects were suggested to be dependent of the exertion of epigenetic effects across genes controlling neural mechanisms common to autism and schizophrenia [30]. Such findings were corroborated by independent evidence of shared genetic vulnerability between autism, schizophrenia, and cannabis use [31,36], the latter being implicated in increasing the risk for psychosis [44,45] as well as inducing the exogenous disruption of the eCB system [46] with effects on brain function and related behavior [47,48]. Furthermore, a developmental trajectory between autism and schizophrenia was supported by evidence that THC exposure, a valid model of psychosis [49,50], results in schizophrenia-related phenotypes among individuals with severe forms of autism [32,35], possibly by altering genes involved in several neurodevelopmental pathways including both autism- and schizophrenia-related ones [33]. Such kind of psychosis among autism spectrum disorder (ASD) patients appeared to be peculiar in terms of significant affective disturbance in comorbidity [35], expanding previous evidence of a potentially specific subtype of ASD with fewer stereotyped interests/behaviors and prone to comorbid atypical affective psychosis [43]. In addition, in the context of autism-associated genetic susceptibility, THC was found to induce biological alterations resembling those observed in schizophrenia either directly [33] or through the indirect effects that an increased liability for lifetime cannabis use may have in affecting dopamine signaling [31]. This is particularly relevant due to dopamine signaling alteration and manipulation being the cornerstone in schizophrenia pathophysiology and treatment [51]. It is worth noting that CB1 receptors’ activation was also demonstrated to lead to the formation of heterodimers with 5-hydroxy-tryptamine (5-HT)2A receptors, further accounting for the negative cognitive effects of THC, because of their expression and functioning in brain regions specifically being involved in memory impairment [52]. Considering the long-lasting evidence of 5-HT [53] and cognitive [54] abnormalities across the autism–psychosis spectrum, whether they are modulated by the eCB system requires further investigation. Detrimental methylation effects of THC/ cannabis exposure on autism-and neurodevelopment- associated genes controlling social behavior, vocalization behavior, and learning were found in both humans and animals [28,29]. In addition, intergenerational inheritance of such altered methylation patterns was suggested in brain areas, such as the nucleus accumbens [28], which are relevant to schizophrenia pathophysiology and treatment [55]. Similarly, following THC/cannabis exposure, autism candidate genes were found to present with bivalent chromatin markings indicative of inherent vulnerability to subsequent epigenetic disruption [29]. Such findings support the notion that THC/cannabis exposure may have negative effects by intergenerationally altering ASD-related genes in brain areas relevant to psychosis and making such genes more susceptible to subsequent risk of disruption, with implications for the manifestation of psychosis later in life. This is congruent with evidence suggesting that social communication, a trait common to autism and schizophrenia, not only shows a change in its phenotypic manifestation over time but also in its genetic architecture [56], due to de novo genetic alterations occurring in adolescence [57]. This is reflected in social communication problems having an association with autism polygenic risk score (PRS) in childhood that declines with age as well as an association with schizophrenia PRS that is the strongest in late adolescence [58]. Interestingly, psychotic experiences among the general population were found to depend on genetic risk for autism, schizophrenia, and neurodevelopmental disorders more widely, with a critical role of the cannabinoid receptor type 2 gene (CNR2) in increasing the risk of presenting with distressing psychotic experiences. Recent evidence indicates that both genetic and epigenetic changes in the dopamine signaling cascade may modulate the psychotomimetic and neurofunctional effects of THC in otherwise healthy subjects, with implications for the manifestation of THC-induced fear-related brain activation and anxiety-like behavior [59]. However, the role of the eCB system modulation in triggering such behavioral aberrancies in the continuum between autism and psychosis remained to be elucidated.

Despite the frequent research reports that a large set of domains may exhibit diametrically opposite phenotypes in autistic-spectrum versus psychotic-spectrum conditions, with a specific focus on schizophrenia [60], we found little evidence supporting a diametral pattern of aberrancies with reference to the eCB system. A single animal study suggested that THC exposure might ameliorate behavioral disturbances in a genetic model of autism, while inducing such behavioral aberrancies in control groups [25]. Similarly, in a single human study, increasing THC doses of cannabidiol (CBD)-rich medical cannabis was reported to increase the chances of getting a therapeutic effect in patients with severe ASD [32]. Such findings were diametric to the evidence for higher psychosis risk in otherwise healthy subjects exposed to increasing doses of THC [61,62]. The preclinical study also reported an upregulation of the cannabinoid receptor type 2 transcript CB2A in the animal model of autism [25], which was discussed by the authors themselves to contrast it with diametral evidence provided by the same research group for lower CB2 receptor function in schizophrenia [63]. 

It is worth mentioning that autism and psychosis may share both diametral and overlapping aspects, depending on the specific biobehavioral domain. For instance, a recent study conducted in over 3000 subjects with either schizophrenia or autism found overlapping abnormalities encompassing within-domain and inter-domain connectivity, primarily involving default mode, sensori-motor, and cognitive-motor regions, along with common structural changes in grey matter volume and density, in comparison to healthy controls. However, the study also revealed disorder-unique changes mostly involving visual and cognitive control functional networks, potentially accounting for phenotypic differences between common clinical symptoms [64].

Despite being historically classified as distinct disorders, the 5th and more recent revision of the Diagnostic and Statistical Manual of Mental Disorders (DSM-5) has gathered neuropsychiatric conditions with the commonality of childhood onset into the single overarching category of neurodevelopmental disorders (NDD) [1]. This follows the evidence of substantial comorbidity and overlap in terms of neuropsychological and psychopathological manifestations as well as neurobiological and genetic underpinnings across NDD [8,65]. A step further has been proposed in terms of seeing NDD in a neurodevelopmental continuum with disorders that typically emerge in late adolescence and early adulthood, such as affective and non-affective psychoses [9]. Such a paradigmatic shift is supported by at least two strong hypotheses. First, according to the neurodevelopmental hypothesis of schizophrenia, core defining features of the condition are a late phenotypic manifestation of an underlying disorder process that starts well before adolescence and adult life with disturbances occurring early in brain development [66]. Interestingly, childhood neurocognitive as well as developmental impairments in language, attention, emotions, and social and motor function have been found to be associated with schizophrenia PRS [67], thus suggesting potential childhood antecedents of schizophrenia due to a genetic liability [68]. An example par excellence is the 25% risk of developing schizophrenia in individuals carrying highly penetrant rare mutations, such as 22q11 microdeletion, because of which they suffer from ASD-related psychopathology as children [69]. Second, according to the wider neurodevelopmental gradient hypothesis, the higher the psychopathological, cognitive, genetic, and sensorimotor severity, the greater the neurodevelopmental impairment and the earlier the disorder onset, along a continuum of severity with ASD with intellectual disability on one hand and affective psychosis on the other [8]. 

A long-lasting debate around ASD regards its symptomatic trajectories over time. Although patterns of overall improvement have been reported as patients grow up, the most severe forms of ASD seem to maintain a high degree of impairment [70]. NDD (e.g., tic disorders, ADHD) and current or past psychiatric disorders (e.g., major depressive disorder, generalized anxiety disorder) in comorbidity seem to play a role in determining a poorer outcome and the persistence of distressing symptoms in the longer term [71]. Findings collected in the current review align and expand such evidence, indicating that THC-induced eCB system alteration results in phenotypes associated with schizophrenia among patients with severe ASD [32,35].

The findings of this systematic review must be interpreted with consideration of some limitations. Research in the field is still too limited and heterogenous, and further studies are needed to fully address the relevance of the eCB system in the continuity between different clinical phenotypes of ASD and schizophrenia-related phenotypes later in life. Whether disruption of the eCB signaling in ASD results in a higher risk of psychosis remains to be tested, and future longitudinal studies will have to investigate such a hypothesis in properly designed clinical trials. Moreover, any other risk factor interacting with the eCB system in conferring risk of psychosis among ASD patients is still to be explored. 

## 5. Conclusions

Evidence provided in this review presents clinical implications. First, a developmental perspective of psychiatric disorders must be implemented to consider age-at-onset and changes over time as well as different developmental periods when interpreting clinical symptoms. More specifically, keeping in mind the evidence for a continuity between childhood psychopathology and psychiatric disorders in adulthood more generally [72], a disease progression between ASD and schizophrenia should be considered. Second, the ‘at-risk mental state’ concept, that has been represented as a milestone in the development of a preventive approach to psychosis and subsequently mental distress more widely [73], should consider the role of ASD in conferring risk for psychosis. Third, youth mental health services for people at risk of psychosis [74] might need to be implemented to intercept people with ASD who may be in the earlier stages of psychosis, adopting multidisciplinary collaborations between different specialized professionals in an enhanced and integrated service of extended primary care [75]. Fourth, ASD treatment should be offered from a preventative perspective to support protective factors and control environmental determinants that may differentially modulate gene expression and stress response, with enduring health effects [76]. Fifth, based on the role of the eCB system disruption in the continuity between ASD and schizophrenia, policy implications of the evidence on cannabis and psychosis [77] should contemplate ASD, discouraging young adults with ASD from using cannabis and informing them of the higher mental health risk of cannabis use, especially in terms of psychosis.

## Figures and Tables

**Table 1:** (**a**). Summary of animal studies investigating ASD and psychosis as a function of the eCB system. (**b**). Summary of human studies investigating ASD and psychosis as a function of the eCB system.

(a)							
Study (Country)	Aim of Study	Type of Study	Population	N	Outcome Measure (Test Name or Description)	ASD and Psychosis as a Function of the eCB System: Summary of Evidence	ASD and Psychosis as a Function of the eCB System: Underlying Model
Onaivi et al., (2011) (USA) [25]	1. To assess THC-induced behavioral changes and 2. eCB system-related gene expression in ASD mice	1. In vivo exposure in animals; 2. Quantitative tissue assessment in animals.	1. Effects of THC: (a) VHI; (b) THC 1 mg/kg; (c) THC 10 mg/kg; 2. CB2 gene expression: non-injected mice.	X	1. Behavior (MFT, FST); 2. Molecular assessment (Neurochemical Analysis of DA, 5HT, and their metabolites, RNA isolation, RT-PCR)	1. MFT, spontaneous wheel running: (a) BTBR males > C57 males; (b) BTBR group: THC (10 mg/kg) < VHI, THC (1 mg/kg); (c) VHI groups: BTBR > C57 > S129; (d) THC (10 mg/kg) groups: BTBR < C57, S129; 2. FST: (a) basal immobility time: BTBR < C57, S129; (b) basal immobility counts: BTBR > C57, S129; (c) BTBR group immobility time: VHI vs. THC (1, 10 mg/kg), NS; THC (1 mg/kg) vs. THC (10 mg/kg), NS; (f) BTBR group immobility count: VHI vs. THC (1, 10 mg/kg), NS; THC (1 mg/kg) vs. THC (10 mg/kg), NS; (g) THC (1, 10 mg/kg) did not modify the immobility time and counts of BTBR vs. C57 and S129; 3. CB2A gene expression in BTBR mice: cerebellum ↑; fontal cortex, striatum, NOCHG.	Diametral
Anderson et al., (2015) (USA) [26]	To assess β-neurexin KO effect on eCB signaling in mice	1. Quantitative brain assessment in animals; 2. In vitro measurement in animals.	cKO mice: 1. Cre; 2. ΔCre.	1. Number of mice: 3–11 per experimental condition; 2. Number of neurons: 8–55 per experimental condition	1. Molecular assessment (RNA isolation, qRT-PCR, in vivo infections, Stereotactic injections, Ca++ imaging); 2. Behavior (OFT, Fear Conditioning); 3. Electrophysiology	1. β-neurexin KO is associated with both ASD and SCZ; 2. β-neurexin KO leads to endocannabinoid-mediated inhibition of synaptic transmission and blocks LTP; 3. β-neurexins KO in CA1-region neurons impairs contextual fear memory; 4. LTP is restored by CB1 inhibition or 2-AG synthesis inhibition.	Overlapping
Doenni et al., (2016) (Canada) [27]	1. To assess adolescent social behavior and 2. eCBs/Aes brain levels following early inflammation in rats	1. In vivo exposure in animals; 2. Quantitative brain assessment in animals.	1. SIT (P40, *n* = 48): (a) LPS; (b) SAL; 2. OFT (P40, *n* = 32): (a) LPS; (b) SAL; 3. CB1 receptor binding (P40, *n* = 20): (a) LPS; (b) SAL; 4. eCB extraction and analysis: (a) P14, *n* = 39; (b) P40, *n* = 34; (c) €, *n* = 4; (d) VHI, *n* = 8; (e) FAAHi, *n* = 8; 5. FAAH activity assay (P40, *n* = 17): (a) LPS; (b) SAL; 6. Oral FAAHi administration (P14, *n* = 32): (a) LPS; (b) SAL; 7. BLA FAAHi injection (P30, *n* = 55): (a) LPS; (b) SAL.	297	1. Molecular assessment (ecBs/AEs brain levels); 2. Behavior (SIT, OFT)	1. LPS injection at P14 leads to impaired social behavior at P40; 2. AEA levels: (a) P14: LPS < SAL; (b) P40: LPS > SAL; 3. 2-AG levels (P14, P40): LPS vs. SAL, NS; 4. CB1 binding site density (P40): LPS < SAL; 5. FAAH activity (P40): LPS > SAL; 6. Oral FAAHi administration ↑ AEA and restores social behavior; 7. BLA FAAHi injection restores social behavior in female.	Overlapping
Schrott et al., (2019) (USA) [28]	To assess cannabis-induced sperm DNA methylation changes and their intergenerational inheritance in rats	1. In vivo exposure in animals; 2. Quantitative tissue assessment in animals.	1. Adults: (a) THC: 7; (b) VHI: 8; 2. Offspring: (a) THC: 6; (b) VHI: 8.	29	Molecular assessment (DNA isolation from sperm, RRBS, DNA and RNA isolation from brain tissue, Bisulphite pyrosequencing, qRT-PCR in brain tissue)	1. DLGAP2 DNA methylation in sperm: THC < VHI; 2. Offspring nucleus accumbens: CpG site 2 hypomethylated as in the sperm of THC exposed adults.	Developmental trajectory
Schrott et al., (2020) (USA) [29]	To assess cannabis-induced sperm DNA methylation changes in rats	1. In vivo exposure in animals; 2. Quantitative tissue assessment in animals.	1. Oral administration: (a) THC: 9; (b) VHI: 8; 2. SC injection: (a) THC: 8; (b) VHI: 7.	32	Molecular assessment (DNA isolation from sperm, RRBS, Bisulphite conversion, Bisulphite pyrosequencing, qRT-PCR)	1. Lrrtm4 DNA methylation in sperm: THC > VHI; 2. Shank1, Syt3, Nrxn1, Nrxn3, Dlg4, Grid1 DNA methylation in sperm: THC < VHI.	Developmental trajectory
Wanner et al., (2020) (USA) [30]	To assess CBD-induced brain DNA methylation changes in mice	1. In vivo exposure in animals; 2. Quantitative brain assessment in animals; 3. Gene-based study in animals.	1. CBD; 2. VHI	X	Molecular assessment (DNA isolation, Bisulphite conversion, RRBS, DMLs, and DMRs detection)	1. CBD administration induces methylation changes in adult mouse hippocampus; 2. ASD [Dlgap4 (3), Shank3 (3), Cadps2 (2), Arid1b (1), Camk2a (1), Lrfn2 (1), Prex1 (1), Shank2 (1), Tsc1 (1), Wdfy3 (1)] and SCZ [Nr4a2 (3), Shank3 (3), Srgap3 (2), Magi2 (1), Tcf4 (1)] are among the top 10 DO terms organized by DMLs and average DMLs/gene.	Overlapping
**(b)**							
**Study (Country)**	**Aim of Study**	**Type of Study**	**Population**	**N**	**Outcome Measure (Test Name or Description)**	**ASD and Psychosis as a Function of the eCB System:** **Summary of Evidence**	**ASD and Psychosis as a Function of the eCB System: Underlying Model**
Stringer et al., (2016) (Netherlands) [31]	To identify genetic risk variants related to lifetime CU	Gene-based study in humans	International Cannabis Consortium: 13 samples from Europe, USA and Australia	32 330	Genetic associations (GWAS)	Association with lifetime cannabis use: 4 genes, 1 intergenic noncoding RNA region: 1. NCAM1: part of the NTAD cluster, involved in neurogenesis and dopaminergic neurotransmission; 2. CADM2: part of the SynCAM family, associated with ASD; 3. SCOC: regulation of gene expression through regulation of transcription factor binding; 4. KCNT2: potassium voltage-gated channel.	Developmental trajectory/Overlapping
Aran et al., (2018) (Israel) [32]	To assess tolerability and efficacy of CBD-rich cannabis in ASD	In vivo treatment exposure in humans	Severe ASD patients	60	Behavior (CaGIC scale, HSQ-ASD, APSI)	1. Behavior improvement in 61% of patients; 2. In 29 insufficient responders, lower CBD:THC ratios (up to 6:1) led to a behavior improvement; 3. Higher CBD:THC ratio (up to 20:1) was well tolerated; 4. Lower CBD:THC ratio led to a serious psychotic episode requiring treatment with an antipsychotic.	Developmental trajectory/Diametral/Overlapping
Guennewig et al., (2018) (Australia) [33]	To assess THC-induced gene alteration in hiPSC-derived neurons	1. In vitro treatment exposure in humans; 2. Gene-based study in humans.	1. Untreated; 2. Acute THC; 3. Chronic THC.	X	1. Molecular assessment (RNA-sequencing, qRT-PCR); 2. Genetic associations (DEGs, enrichment analysis)	1. Acute THC exposure: 497 altered genes; 2. Chronic THC exposure: 810 altered genes; 3. High overlap: subsets of genes involved in glutamate receptor pathway and mitochondrial function; 4. THC-altered transcripts: 80 genes linked to ASD, fewer genes linked to SCZ; 5. Overlap between THC and SCZ: WNT and mitochondrial signaling pathways.	Developmental trajectory
Legge et al., (2019) (Netherlands) [34]	1. To assess shared genetic liability and 2. identify genetic loci associated with PEs	Gene-based study in humans	UK Biobank individuals: 1. MHQ; 2. nMHQ.	127,966	Genetic associations (GWAS, genetic correlation, PRSs, CNV)	1. PRSs: PEs associated with genetic liability for SCZ, ASD; 2. CNV: burden of SCZ- and NDDs-related CNV in individuals reporting PEs; 3. GWAS of any PEs: ANK3 intronic variant (rs10994278); 4. GWAS of distressing PEs: CNR2 (encoding for CB2) intronic variant (rs75459873), not associated with CU.	Developmental trajectory
Schrott et al., (2019) (USA) [28]	To assess cannabis-induced sperm DNA methylation changes and their intergenerational inheritance	1. Gene-based study in humans; 2. Quantitative tissue assessment in humans.	1. Gene-based tests on sperm: (a) Users: 12; (b) Non-users: 12; 2. Brain tissue assessment: 28; 3. Testis tissue assessment: 3.	55	Molecular assessment (DNA isolation from sperm, RRBS, DNA, and RNA isolation from conceptal brain and testis tissue, Bisulphite pyrosequencing, qRT-PCR in conceptal brain tissue)	1. DLGAP2 dysregulation: associated with ASD and SCZ; 2. DLGAP2 DNA methylation in sperm: Cannabis-users < Non Cannabis-users; 3. Inverse relationship between DLGAP2 DNA methylation in brain and mRNA expression (female > male).	Developmental trajectory
Schrott et al., (2020) (USA) [29]	To assess cannabis-induced sperm DNA methylation changes	1. Gene-based study in humans; 2. Quantitative tissue assessment in humans.	Gene-based tests on sperm: 1. Users: 12; 2. Non-users: 12	24	Molecular assessment (DNA isolation from sperm, RRBS, Bisulphite conversion, Bisulphite pyrosequencing, qRT-PCR)	1. Syt3, Lrrtm4, Nrxn1, Nrxn3, Shank1, Dlg4, Grid1 genes major Biological Process GO terms: social behavior, vocalization behavior, learning; 2. Bivalent chromatin marks: THC alters genes connected to ASD, which also present with future risk of disruption.	Developmental trajectory
Al-Soleiti et al., (2021) (Netherlands) [35]	To assess THC-induced psychotic symptoms in ASD	In vivo exposure in humans	ASD patients	3	Clinical assessment	1. ‘self-prescribed’ medical cannabis (sativa/indica mixtures, 20 % THC, 0 % CBD) to relieve anxiety → hallucinations and paranoid delusions, mood swings → induced BIP I, mixed state, with psychotic symptoms; 2. diagnosis of ARMS at 17 → marijuana consumption to feel calmer (1 g per day) → intense auditory hallucinations, paranoia → diagnosis of SCZ at 19 → medical marijuana card (3–4 g per day, indica strains 10 % THC, occasionally marijuana wax with 90 % THC) → increasing psychotic symptoms.	Developmental trajectory
Powell et al., (2021) (USA) [36]	To assess iDANs SEGs enrichment for psychiatric diseases	1. Quantitative brain assessment in humans; 2. In vitro measurement in humans; 3. Gene-based study in humans.	1. iDANs; 2. iGANs; 3. iGLUTs	X	1. Molecular assessment (RNA isolation, RT-qPCR, Immunocytochemistry, FANS, nuclear RNA-sequencing on brain samples, whole RNA-sequencing on in vitro samples, MEA, Dopamine ELISA); 2. Electrophysiology.	1. SEGs in iDANs are enriched for CUD, ASD and SCZ; 2. CUD and SCZ risk loci are enriched for unique subsets of SEGs in iDANs, iGANs, and iGLUTs; 3. ASD risk loci are only enriched in iDAN SEGs.	Overlapping

ASD, Autism Spectrum Disorder; eCB, endocannabinoid; THC, Δ9-tetrahydrocannabinol; VHI, vehicle; mg/kg, milligrams per kilogram; MFT, Motor Function Test; FST, Forced Swim Test; DA, dopamine; 5HT, serotonin; RNA, Ribonucleic Acid; RT-PCR, Reverse transcription polymerase chain reaction; BTBR, BTBR T+tf/J mice; C57, C57BL/6 mice; S129, 129S1/SvImJ mice; vs., versus; NS, not significant; CB2A, CNR2 Gene; NOCHG, no change; cKO, conditional triple KO mice; Cre, infected with lentiviruses to delete β-neurexin; ΔCre, truncate Cre-ricombinase; qRT-PCR, Real-Time Quantitative Reverse Transcription PCR; OFT, Open-Fielt Test; LTP, Long-Term Plasticity; CB1, Cannabinoid receptor type 1; 2-AG, 2-Arachidonoylglycerol; AEs, acylethanolamines; SIT, Social Interaction Test; P40, Postnatal day 40; LPS, lipopolysaccharide; SAL, saline; P14, Postnatal day 14; FAAHi, FAAH inhibitor PF-04457845; FAAH, fatty acid amide hydrolase; P30, Postnatal day 30; AEA, anandamide; BLA, basolateral amygdala; DNA, Deoxyribonucleic Acid; RRBS, reduced representation bisulphite sequencing; DLGAP2, DLG Associated Protein 2; SC, subcutaneous; Lrrtn4, Leucine Rich Repeat Transmembrane Neuronal 4; Shank1, SH3 And Multiple Ankyrin Repeat Domains 1; Syt3, Synaptotagmin 3; Nrxn1, Neurexin 1; Nrxn3, Neurexin 3; Dlg4, Discs Large MAGUK Scaffold Protein 4; Grid1, Glutamate Ionotropic Receptor Delta Type Subunit 1; DMLs, Differentially methylated loci; DMRs, Differentially methylated regions; CBD, cannabidiol; Dlgap4, DLG Associated Protein 4; Cadps2, Calcium Dependent Secretion Activator 2; Arid1b, AT-Rich Interaction Domain 1B; Camk2a, Calcium/Calmodulin Dependent Protein Kinase II Alpha; Lrfn2, Leucine Rich Repeat And Fibronectin Type III Domain Containing 2; Prex1, Phosphatidylinositol-3,4,5-Trisphosphate Dependent Rac Exchange Factor 1; Shank2, SH3 And Multiple Ankyrin Repeat Domains 2; Tsc1, TSC Complex Subunit 1; Wdfy3, WD Repeat And FYVE Domain Containing 3; SCZ, schizophrenia; Nr4a2, Nuclear Receptor Subfamily 4 Group A Member 2; Srgap3, SLIT-ROBO Rho GTPase Activating Protein 3; Magi2, Membrane Associated Guanylate Kinase, WW And PDZ Domain Containing 2; Tcf4, Transcription Factor 4; DO, Disease Ontology; CU, cannabis use; GWAS, Genome-Wide Association Studies; NCAM1, Neural Cell Adhesion Molecule 1; CADM2, Cell Adhesion Molecule 2; SCOC, Short Coiled-Coil Protein; KCNT2, Potassium Sodium-Activated Channel Subfamily T Member 2; CaGIC, Caregiver Global Impression of Change; HSQ-ASD, Home Situation Questionnaire-Modified for ASD; APSI, Autism Parenting Stress Index; hiPSC, Human Induced Pluripotent Stem Cell; DEGs, Differentially Expressed Genes; ID, intellectual disability; PEs, psychotic experiences; MHQ, Mental Health Questionnaire; nMHQ, individuals who provided a negative response to all psychotic experience symptom questions at MHQ; PRSs, Polygenic Risk Scores; CNV, Copy Number Variation; NDDs, Neurodevelopmental Disorders; ANK3, Ankyrin 3; ARMS, At Risk Mental State; iDANs, induced dopaminergic neurons; SEGs, Specifically Expressed Genes; iGANs, induced GABAergic neurons; iGLUTs, induced glutamatergic neurons; FANS, fluorescence-activated nuclei-sorting; MEA, Multielectrode array; ELISA, Enzyme-linked immunosorbent assay; CUD, Cannabis Use Disorder; ↑, increased; >, higher than; <, lower than.

**Table 2:** (**a**). Methodological quality of animal studies investigating ASD and psychosis as a function of the eCB system. (**b**). Methodological quality of human studies investigating ASD and psychosis as a function of the eCB system.

(a)										
Study	Study Design	Defined Study Population	Age	Gender	Control	eCB System Involvement	ASD Involvement	Psychosis Involvement	Statistical Analyses	Funding or Sponsorship
Onaivi et al., (2011) (USA) [25]	√ Analytic, observational, interventional	√ BTBR, C57, S129 mice	√ Adult	√ Male and female	√ VHI; C57; S129	√ 1. THC single administration: (a) 1 mg/kg IP; (b) 10 mg/kg IP; 2. CB2 gene expression	√ Idiopathic animal model	√ Behavioral features	√ Student’s *t*-test; ANOVA; Tukey’s test	√
Anderson et al., (2015) (USA) [26]	√ Analytic, observational	√ NBF mice	√ 1. mRNA measurements: P30;2. Neuron cultures from newborn NBF: DIV 3–4 to DIV 14–16	X	√ ΔCre	√ eCBs/AEs signaling	√ Genetically induced animal model	√ Genetically induced animal model	√ Student’s *t*-test	√
Doenni et al., (2016) (Canada) [27]	√ Analytic, observational, interventional	√ Sprague Dawley rats	√ P14 and P40	√ Male and female	√ SAL	√ 1. Double eCBs/AEs levels assessment (P14, P40); 2. FAAHi single administration (oral, BLA injection)	√ Inflammatory-induced animal model; Behavioral features	√ Inflammatory-induced animal model; Behavioral features	√ F-test; Bonferroni’s post hoc test; Student’s *t*-test; ANOVA	√
Schrott et al., (2019) (USA) [28]	√ Analytic, observational, interventional	√ Sprague Dawley rats	√ 9 weeks	√ Male	√ VHI	√ THC daily administration: 4 mg/kg SC, 28 days	√ Genetic liability	√ Intergenerational genetic liability	√ Student’s *t*-test; Bonferroni’s post hoc test; Pearson correlation	√
Schrott et al., (2020) (USA) [29]	√ Analytic, observational, interventional	√ Sprague Dawley rats	√ Young adult	√ Male	√ VHI	√ THC daily administration: (a) 2 mg/kg oral, 12 days; (b) 4 mg/kg SC, 28 days	√ Genetic liability	√ Genetic liability	√ Student’s *t*-test; Bonferroni’s post hoc test; Pearson correlation; Fisher’s exact test	√
Wanner et al., (2020) (USA) [30]	√ Analytic, observational, interventional	√ C57 mice	√ 14 weeks	√ Male	√ VHI	√ CBD daily administration: 20 mg/kg oral, 14 days	√ Genetic liability	√ Genetic liability	√ chi-square test; Fisher’s exact test; Benjamini-Hochberg adjusted *p*-values	√
**(b)**										
**Study**	**Study Design**	**Defined Study Population**	**Age**	**Gender**	**Control**	**eCB System Involvement**	**ASD Involvement**	**Psychosis Involvement**	**Statistical Analyses**	**Funding or Sponsorship**
Stringer et al., (2016) (Netherlands) [31]	√ Meta-analysis	√ Lifetime cannabis use	√ 16–87 years (average 34 years)	√ Male and female (30–66%)	√ 4 independent replication samples	√ Cannabis exposure	√ Genetic liability	√ Genetic liability	√ Logistic regression	√
Aran et al., (2018) (Israel) [32]	√ Analytic, observational, interventional	√ DSM-5 (77% low cognitive functioning according to ADOS or CARS)	√ 5–17 years [11.8 (± 3.5)]	√ Male (83%) and female	X	√ CBD-rich treatment (CBD:THC = 20:1, sublingual administration), 2–3 times per day, up to 10 mg/kg/die	√ Diagnosed patients	√ Adverse event	√ Mann–Whitney U test; Spearman’s rho correlation; Pearson correlation	X
Guennewig et al., (2018) (Australia) [33]	√ Analytic, observational, interventional	√ General population: hiPSC-derived neurons	X	X	√ Untreated; SCZ hiPSC- derived neurons	√ 1. (a) Acute THC-exposure (1 μM for 24 h); (b) Chronic THC-exposure (50 nM; 5 treatments over 7 days); 2. Genetic liability	√ Genetic liability	√ Genetic liability; SCZ-like biological alterations	√ ANOVA; Tukey’s test for multiple comparisons	√
Legge et al., (2019) (Netherlands) [34]	√ Analytic, observational	√ MHQ	√ 64 (± 7.6) years	√ Male (44%) and female	√ nMHQ	√ Genetic liability	√ Genetic liability	√ 1. Psychotic symptoms; 2. Genetic liability	√ Logistic regression; Bonferroni’s correction	√
Schrott et al., (2019) (USA) [28]	√ Analytic, observational	√ General population: 1. Screened for (a) past 6-month CU; (b) UDS results; (c) [THCCOOH] in urine;2. Conceptal tissues from elective pregnancy termination	√ 1. 18–40 years; 2. 67–122 gestational days	√ Male and female	√ Gene-based tests on sperm: Non-users	√ Cannabis exposure	√ Genetic liability	√ Genetic liability	√ Student’s *t*-test; Bonferroni’s post hoc test; Pearson correlation	√
Schrott et al., (2020) (USA) [29]	√ Analytic, observational	√ General population: screened for 1. past 6-month CU; 2. UDS results; 3. [THCCOOH] in urine	√ 18–40 years	√ Male	√ Non-users	√ Cannabis exposure	√ Genetic liability	√ Genetic liability	√ Student’s *t*-test; Bonferroni’s post hoc test; Pearson correlation; Fisher’s exact test	√
Al-Soleiti et al., (2021) (Jordan) [35]	√ Case report	√ DSM-5	√ 1. 20 years old; 2. 23 years old; 3. 23 years old	√ Male	X	√ 1. 2-months daily CBD oil (<0.03 % THC); 2. Self-prescribed sativa/indica mixtures (20 % THC); 3. Marijuana consumption (until 90% THC)	√ Diagnosed patients	√ Adverse event	X	X
Powell et al., (2021) (USA) [36]	√ Analytic, observational	√ General population: 1. hiPSC derived iDANs; 2. Post-mortem samples	√ Post-mortem samples: adult brains	X	√ hiPSC derived 1. iGANs; 2. iGLUTs	√ Genetic liability for CUD	√ Genetic liability	√ Genetic liability	√ ANOVA; Tukey’s test for multiple comparisons; Bonferroni’s post hoc test	√

eCB, endocannabinoid; ASD, Autism Spectrum Disorder; BTBR, BTBR T+tf/J mice; C57, C57BL/6 mice; S129, 129S1/SvImJ mice; VHI, vehicle; THC, Δ9-tetrahydrocannabinol; mg/kg, milligrams per kilogram; IP, intraperitoneal; ANOVA, analysis of variance; NBF, Neurexin β-floxed; P30, Postnatal day 30; DIV, Days in vitro; ΔCre, truncate Cre-ricombinase; AEs, acylethanolamines; P14, Postnatal day 14; P40, Postnatal day 40; SAL, saline; FAAHi, FAAH inhibitor PF-04457845; BLA, basolateral amygdala; SC, subcutaneous; CBD, cannabidiol; DSM-5, The Diagnostic and Statistical Manual of Mental Disorders, Fifth Edition; ADOS, Autism Diagnostic Observation Schedule; CARS, Childhood Autism Rating Scale; hiPSC, Human Induced Pluripotent Stem Cell; SCZ, schizophrenia; μM, micromolar; h, hour/hours; nM, nanomolar; MHQ, Mental Health Questionnaire; nMHQ, individuals who provided a negative response to all psychotic experience symptom questions at MHQ; UDS, Urine Drugs Screening; THCCOOH, carboxy-Δ9-tetrahydrocannabinol; iDANs, induced dopaminergic neurons; SEGs, Specifically Expressed Genes; iGANs, induced GABAergic neurons; iGLUTs, induced glutamatergic neurons; CUD, Cannabis Use Disorder; <, lower than.

## Data Availability

Not applicable.
